# Supplementary material for: Cryptic bioactivity capacitated by synthetic hybrid plant peptides
Source: Nat Commun. 2017 Feb 6;8:14318. doi: 10.1038/ncomms14318 (PMC5303819; doi:10.1038/ncomms14318)
Supplement: Supplementary Information — Supplementary Figures [file ncomms14318-s1.pdf]

Supplementary Figure 1

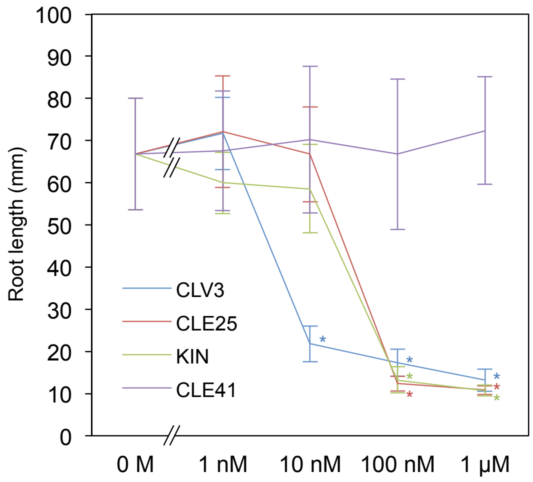

Supplementary Figure 1. Dose-response relationships of CLE activities on root growth.
Effects of various concentrations of CLE peptides on 14 day-old root length. Data represent mean values ± s.d. with asterisks indicating a significant difference from mock treatment (0M) in two-tailed Weltch's *t*-test (*p<0.05, n = 12–14, see Supplementary Data 1 for individual sample sizes).

Supplementary Figure 2

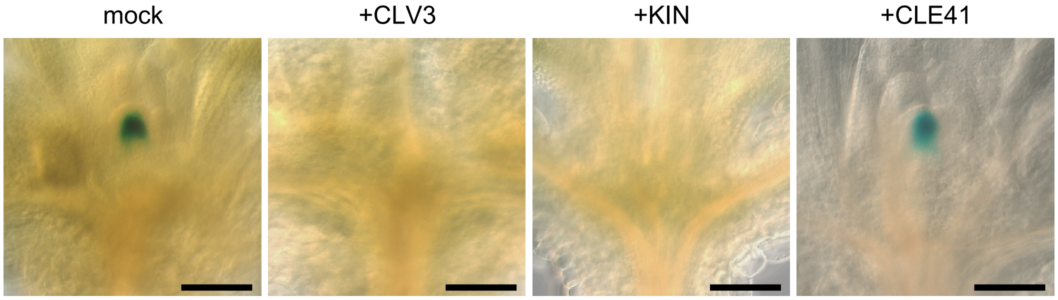

Supplementary Figure 2. Effects of peptides on *WUSprom:GUS*.
GUS-stained shoot apices of *WUSprom:GUS* plants grown for 10 days with 10 μM peptides indicated above. Scale bars, 100 μm. Representative photos among four independent samples are shown.

Supplementary Figure 3

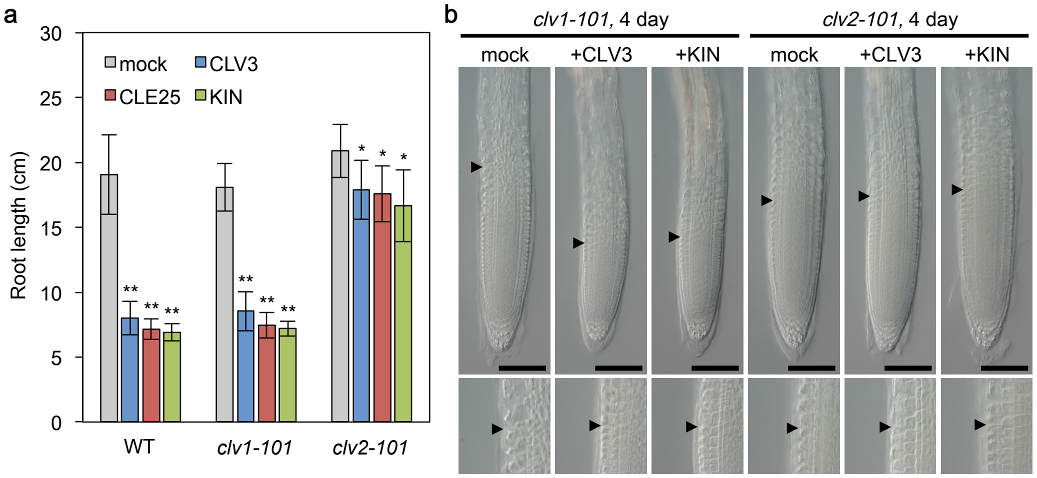

Supplementary Figure 3. Effects of peptides on mutant root growth.
(a) Root length for WT (blue), *clv1-101* (red) and *clv2-101* (green) plants grown for 7 days with 1 μM peptides. Note that the root growth of *clv2-101* shows significant resistance to the peptide treatments. Data represent mean values ± s.d. with asterisks indicating significant differences from mock treatment (grey) in each genetic background in two-tailed Weltch's *t*-test (*$p<10^{-4}$, **$p<10^{-10}$, n = 11–16, see Supplementary Data 1 for individual sample sizes). (b) Effects of 1 μM peptides on 4 day-old RAM morphology. The arrowheads indicate the RAM areas. Lower panels show magnification of the boundary areas. Scale bars, 100 μm. Representative photos among four or more independent samples are shown.

Supplementary Figure 4

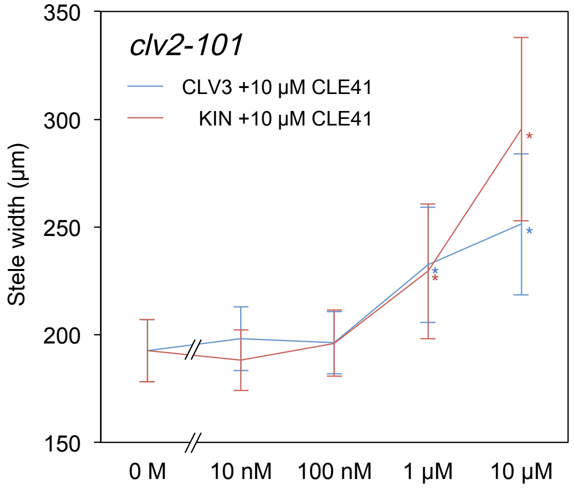

Supplementary Figure 4. Dose-response relationships of the synergistic CLE activities on *clv2* mutant stele thickening. Effects of various concentrations of CLV3 and KIN on stele width were examined in 10 day-old *clv2-101* mutants treated simultaneously with 10 μM CLE41. Data represent mean values ± s.d. with asterisks indicating a significant difference from single CLE41 treatment (indicated as 0 M) in two-tailed Welch's *t*-test (*$p<0.05$, n = 15–16, see Supplementary Data 1 for individual sample sizes).

Supplementary Figure 5

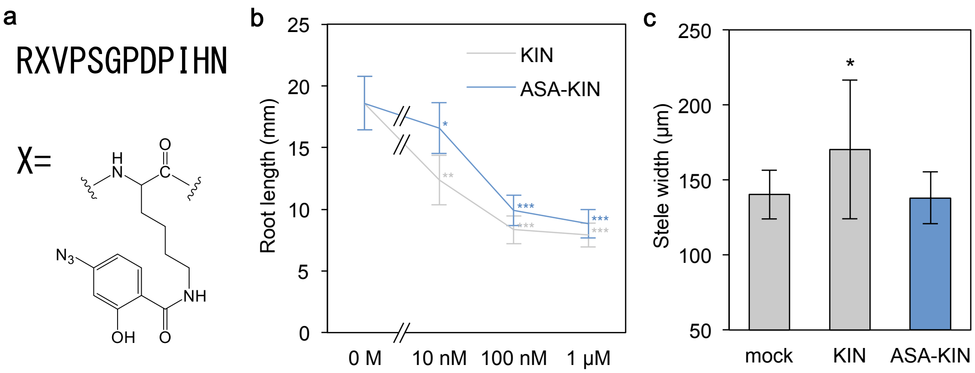

Supplementary Figure 5. Bioactivities of ASA-KIN peptide.
(a) Structure of the ASA-KIN peptide. (b) Root-shortening activity of ASA-KIN was slightly reduced compared to KIN in 7 day-old plants. (c) Bioactivity of ASA-KIN in hypocotyl stele thickening assay, compared to KIN in 10 day-old plants. For **b** and **c**, data represent mean values ± s.d. with asterisks indicating significant differences from mock treatment in two-tailed Welch's *t*-test (* $p<10^{-4}$, ** $p<10^{-11}$, *** $p<10^{-16}$, n = 22–24 in **b** and * $p<0.05$, n = 13–15 in **c**, see Supplementary Data 1 for individual sample sizes).

Supplementary Figure 6

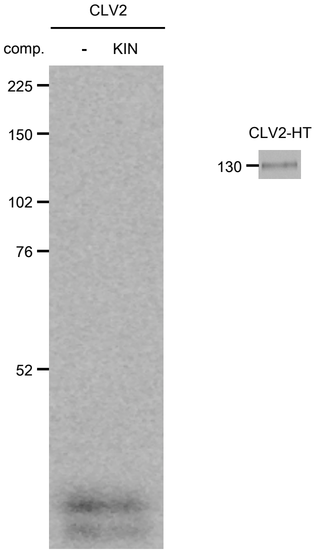

Supplementary Figure 6. Photoaffinity labeling assay between CLV2 and [^{125}I]ASA-KIN.
No signal for [^{125}I]ASA-KIN bound to CLV2 ectodomain was detected. The right panel shows the purified Halo-Tagged CLV2 protein (CLV2-HT) used, visualized by HaloTag TMR reagent.

Supplementary Figure 7

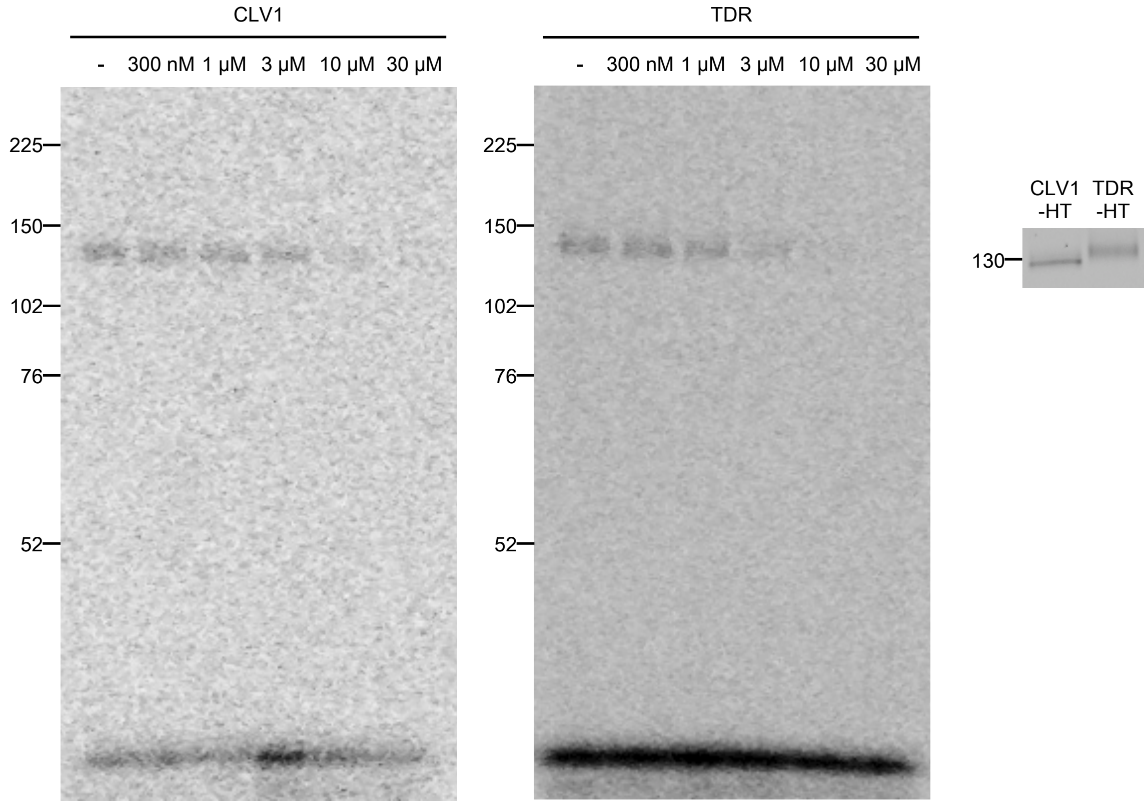

Supplementary Figure 7. Comparison of the binding manners of KIN with CLV1 and TDR.
Competitive displacement of [^{125}I]ASA-KIN binding to CLV1 and TDR ectodomains with various concentrations of unlabeled KIN peptide. The rightmost panel shows the purified Halo-Tagged receptors (CLV1-HT, TDR-HT) used, visualized by HaloTag TMR reagent.

Supplementary Figure 8

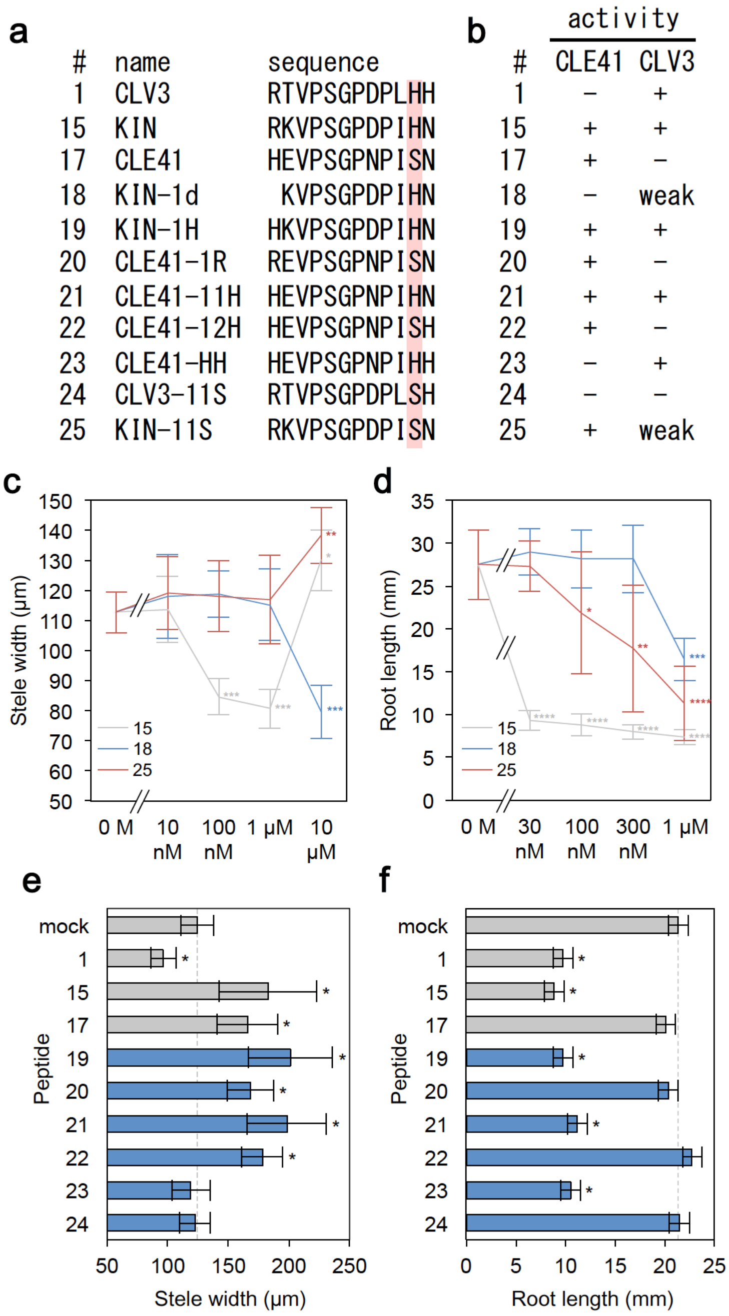

Supplementary Figure 8. Contribution of individual residues to CLE activities.
(a) Sequence alignment of CLE peptides. The 11th residue, important for CLV3-type activity, is colored red. (b) Summary of the results in **c** to **f**. CLV3- or CLE41-type CLE activity was observed (+), observed weakly (weak), or not observed (-). (c) Effects of various concentrations of peptides on stele thickening of 10 day-old plants. (d) Effects of various concentrations of peptides on root length of 7 day-old plants. (e) Effects of 10 μM peptides on 10 day-old stele width. (f) Effects of 1 μM peptides on 7 day-old root length. For **c** to **f**, data represent mean values ± s.d. with asterisks indicating significant differences from mock treatment in Welch's *t*-test (*$p<10^{-3}$ **$p<10^{-6}$, ***$p<10^{-9}$, n = 12–13 in **c**, *$p<0.05$, **$p<10^{-3}$, ***$p<10^{-6}$, ****$p<10^{-8}$, n = 11–12 in **d**, *$p<10^{-4}$, n = 14–16 in **e** and *$p<10^{-8}$, n = 11–12 in **f**, see Supplementary Data 1 for individual sample sizes). Grey dashed lines indicate the mock treatment level.

Supplementary Figure 9

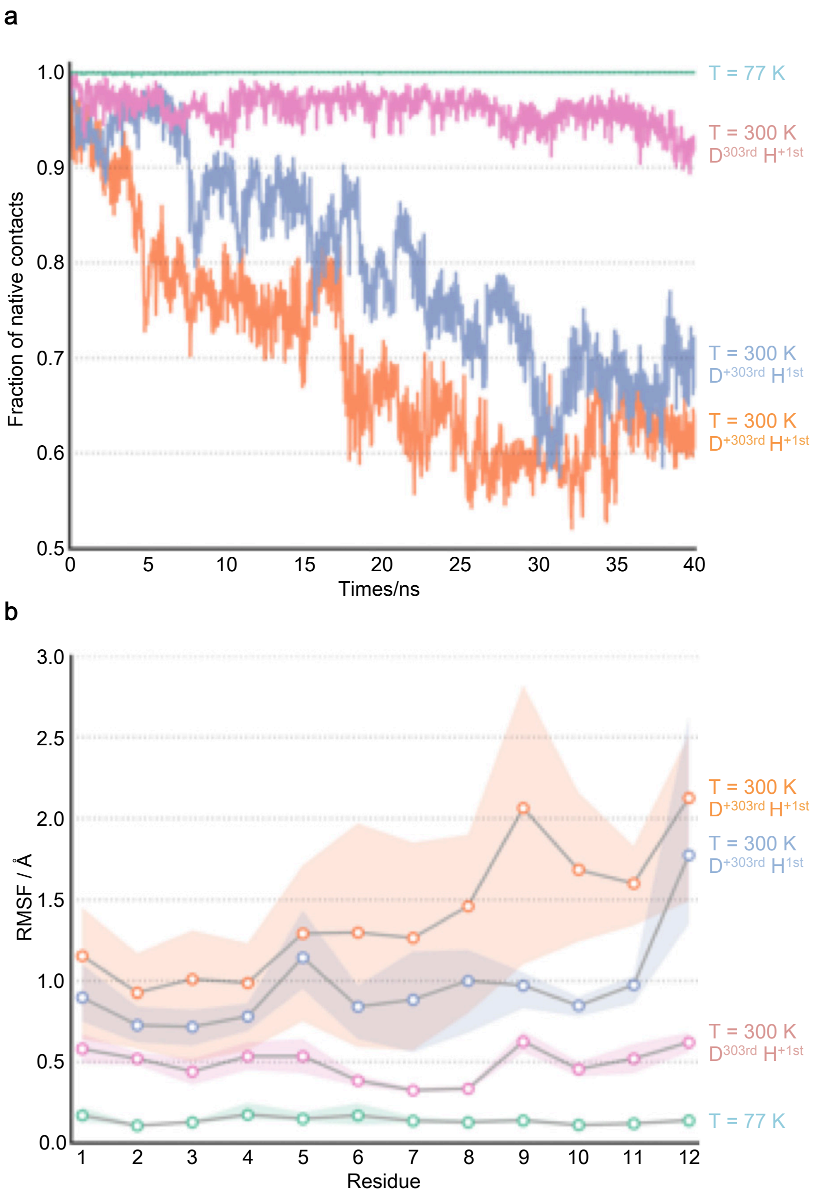

Supplementary Figure 9. Molecular dynamics simulations on the stability of CLE41-TDR interaction. (a) Fraction of native contacts. Distances of heavy atoms between CLE41 and TDR are monitored and compared to the initial time (t = 0, the native conformation). (b) Root mean squared fluctuations (RMSFs) around their mean coordinates of the CLE41's C_{α}-atoms (1st-12th as indicated below) from the entire simulation time. The colored areas in (b) represent maximum and minimum values obtained from three independent simulation trajectories, while the lines represent their averaged values. Different combinations of temperatures and protonation states of CLE41-TDR are examined. Green ("T=77 K"): 77 K, H^{1st} protonated and D^{303rd} protonated. Pink ("D^{303rd} H^{+1st}"): 300 K, H^{1st} protonated, D^{303rd} unprotonated. Blue ("D^{+303rd} H^{1st}"): 300 K, H^{1st} unprotonated, D^{303rd} protonated. Orange ("D^{+303rd} H^{+1st}"): 300 K, H^{1st} protonated and D^{303rd} protonated.

Supplementary Figure 10

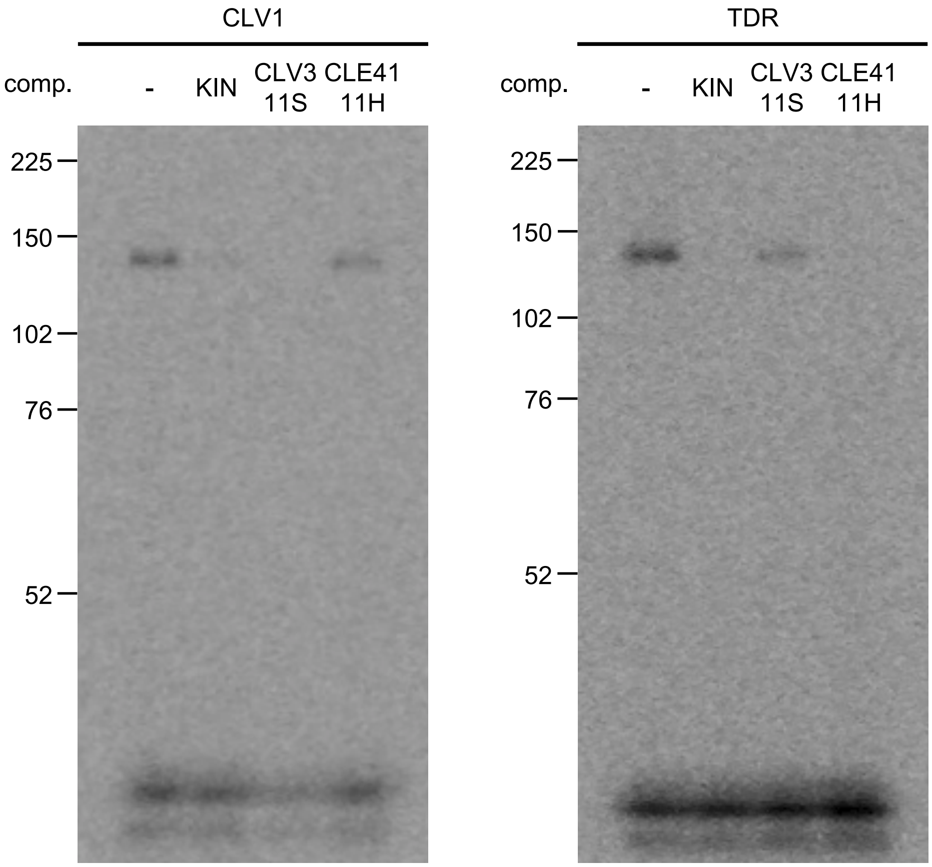

Supplementary Figure 10. Effects of 11th residue substitution on the CLE peptide-receptor interactions. Competitive displacement of [^{125}I]ASA-KIN binding to CLV1 (left) and TDR (right) ectodomains with 1,000-fold excess amount of unlabeled KIN, CLV3-S^{11th} and CLE41-H^{11th} peptides.

Supplementary Note 1

Structure preparation for Molecular Dynamics

The three dimensional coordinates for TDR/CLE41 were obtained from the X-ray structure provided by Zhang et al[1]. The missing coordinates of heavy atoms, in particular those of the unresolved N-terminal residues 61 to 64 were added using the Modeller program[2]. The MD simulations were prepared using tools of the GROMACS simulation package[3-5] using parameters from the ff14SB force field[6]. The pKas of titratable residues were estimated using the PROPKA3.0 program[7]. The protonation states were assigned according to pH 5.5, which is described in more detail below.

The TDR/CLE41 complex was centered in a dodecahedral box with periodic boundary conditions and solvated with TIP3P water molecules[8]. The system was neutralized by the addition of chloride ions. After a short energy minimization to remove bad sterical contacts, the system was equilibrated for 1 ns under constant volume conditions (NVT), restraining the positions of the protein atoms to their original energy minimized positions with a harmonic potential with a force constant of 1000 kJ / (mol nm^{-2}). The temperature was maintained at 300 K or 77 K (the boiling point of liquid nitrogen, used in the crystal preparation for structure determination) using a velocity-rescaling thermostat[9]. After that, the solvent was further equilibrated under constant pressure conditions (NPT) for 1 ns to adjust to a pressure of 1 atm using the Parrinello-Rahman[10,11]. The time constants for

temperature and pressure coupling were 0.1 ps and 2.0 ps, respectively. All bonds were constrained during the simulation with the LINCS algorithm[12], facilitating a timestep of 2 fs. The long-range electrostatic interactions were treated with the particle mesh Ewald method[13]. For each modelled combination of protonation states, three independent trajectories were simulated, starting with identical atom positions, but using different initial atom velocities, drawn randomly from a Maxwell-Boltzmann distribution. Analysis was performed using the MDTraj program package[14]. The fraction of native contacts was calculated according to the Supporting Information in Best et al[15]. The hydrogen bonds were analyzed using the "Wernet-Nilsson" function of MDTraj.

Calculation of pKas of titratable residues

Since electrostatic interactions between CLE41 and TDR potentially change considerably with the number of charged residues at their interface, the assignment of correct protonation states according to their pKa values is imperative for the their simulation. The pKas were estimated using PROPKA3.0 on a webserver (http://nbcr-222.ucsd.edu/pdb2pqr). All histidines were modelled protonated/charged, except His^{207th} and His^{229th} which were modelled neutral. All glutamate residues were modelled unprotonated/charged, except E^{181st}, E^{231st} and E^{555th} which were modelled protonated/neutral. Likewise, all aspartates were modelled unprotonated/charged, except D^{255th} and D^{375th} which were modelled protonated/neutral. H^{1st} of CLE41 and D^{303rd} of TDR were modelled in both their

protonated and unprotonated forms. Therefore, three different combinations of protonation state were modelled: His^{1st} protonated/D^{303rd} protonated, His^{1st} unprotonated/D^{303rd} protonated, and His^{1st} protonated/D^{303rd} unprotonated. The combination His^{1st} unprotonated/D^{303rd} unprotonated was omitted since the simulations with protonated and unprotonated His^{1st} showed similar behavior. The calculated pKas are listed below.

Estimated pKas of titratable residues at the TDR/CLE41 interface. For comparison, the pKas were also calculated with the X-ray structure of only TDR.

	X-ray (TDR/CLE41)	X-ray (only TDR)	Modelled as
H1	5.5	—	protonated and unprotonated
H207	4.8	5.3	unprotonated
H229	3.8	5.9	unprotonated
E181	5.6	5.4	protonated
E231	6.7	4.0	protonated
E555	7.3	4.6	protonated
D255	7.3	5.1	protonated
D303	6.2	5.2	protonated and unprotonated
D375	7.4	5.5	protonated

Supplementary references

1. Zhang, H., Lin, X., Han, Z., Qu, L.J. & Chai, J. Crystal structure of PXY-TDIF complex reveals a conserved recognition mechanism among CLE peptide-receptor pairs. *Cell Res.* **26**, 543–555 (2016).

2. Webb, B. & Sali, A. Comparative Protein Structure Modeling Using MODELLER. *Curr. Protoc. Bioinformatics.* **47**, 5.6.1–5.6.32 (2014).

3. Van Der Spoel, D. *et al.* GROMACS: fast, flexible, and free. *J. Comput. Chem.* **26**, 1701–1718 (2005).

4. Pronk, S. *et al.* GROMACS 4.5: a high-throughput and highly parallel open source molecular simulation toolkit. *Bioinformatics.* **29**, 845–854 (2013).

5. Abraham, M.J. *et al.* GROMACS: High performance molecular simulations through multi-level parallelism from laptops to supercomputers. *SoftwareX.* **1–2**, 19–25 (2015).

6. Maier, J.A. *et al.* ff14SB: improving the accuracy of protein side chain and backbone parameters from ff99SB. *J. Chem. Theory Comput.* **11**, 3696–3713 (2015).

7. Olsson, M.H. *et al.* PROPKA3: consistent treatment of internal and surface residues in empirical pKa predictions. *J. Chem. Theory Comput.* **7**, 525–537 (2011).

8. Jorgensen, W.L. *et al.* Comparison of simple potential functions for simulating liquid water. *J. Chem. Phys.* **79**, 926–935 (1983).

9. Bussi, G., Donadio, D. & Parrinello, M. Canonical sampling through velocity rescaling. *J Chem Phys.* **126**, 014101 (2007).

10. Parrinello, M. & Rahman, A. Polymorphic transitions in single crystals: A new molecular dynamics method. *J. Appl. Phys.* **52**, 7182–7190 (1981).

11. Nosé, S. & Klein, M.L. Constant pressure molecular dynamics for molecular systems. *Mol. Phys.* **50**, 1055–1076 (1983).

12. Hess, B. P-LINCS: A parallel linear constraint solver for molecular simulation. *J. Chem. Theory Comput.* **4**, 116–122 (2008).

13. Darden, T., York, D. & Pedersen, L. Particle mesh Ewald: An N□ log (N) method for Ewald sums in large systems." *J. Chem. Phys.* **98**, 10089–10092 (1993).

14. McGibbon, R.T., Beauchamp, K.A., Harrigan, M.P., Klein, C., Swails, J.M., Hernández, C.X., Schwantes, C.R., Wang, L.P., Lane, T.J. & Pande, V.S. MDTraj: A Modern Open Library for the Analysis of Molecular Dynamics Trajectories. *Biophys. J.* **109**, 1528–1532 (2015).

15. Best, R. B., Hummer, G., & Eaton, W. A. Native contacts determine protein folding mechanisms in atomistic simulations. *Proc.Natl. Acad. Sci.*, **110**, 17874–17879 (2013).
